# Behavioral Patterns of Depression Patients and Control Population

**DOI:** 10.3390/ijerph19159506

**Published:** 2022-08-02

**Authors:** María Carreira-Míguez, Eduardo Navarro-Jiménez, Vicente Javier Clemente-Suárez

**Affiliations:** 1 Faculty of Sports Sciences, Universidad Europea de Madrid, Tajo Street s/n, 28670 Madrid, Spain; mcarreiram@hotmail.com; 2 Facultad de Ciencias de la Salud, Universidad Simón Bolívar, Barranquilla 080002, Colombia; enavarro27@unisimonbolivar.edu.co; 3 Grupo de Investigación en Cultura, Educación y Sociedad, Universidad de la Costa, Barranquilla 080002, Colombia

**Keywords:** depression, nutrition, physical activity, dental pathology, oral health, questionnaire

## Abstract

Behavioral and multifactorial factors, such as psychological, nutritional, dental pathology, and physical activity habits, are factors that control depression. The objective of the present study was to analyze the differences in the behavioral, psychological, nutritional, dental pathology, and physical activity patterns of the depressed and control population. Forty-eight participants with depression (45.7 ± 12.0) and one hundred participants in a control group without any pathology or medication (48.9 ± 7.9) were interviewed using an online questionnaire. The multifactorial items of psychology, oral behavior, nutritional habits, and physical activity profile were analyzed through a set of questionnaires. The results showed how the depression group showed significantly higher psychological measures related to personality, anxiety, depression, loneliness, perceived stress, and psychological inflexibility than the control group. The control group showed significantly higher weekly vitality, vitality at the end of the week, weekly frequency of juice, wine, coffee, fresh vegetable salad, and Bristol scale than the depression group. Higher values of migraine headache, weekly soft drink frequency, and digestion after meals were found in the depression group. In addition, patients with depression also presented poor dental health, presenting higher rates of gastritis or heartburn, dry mouth, dental sensitivity, and sick days per year than the control group. Both groups presented a pattern of low physical activity. This information allows a better understanding of a multifactorial disease, as well as the creation of intervention and prevention protocols for this disease at a behavioral and lifestyle level.

## 1. Introduction

Depression is a common disease throughout the world, with an estimated value of 3.8% of the affected population. Approximately 280 million people in the world have depression [1]. The etiology of depression is complex, involving multiple factors, both genetic, biological, and psychosocial. Within biologicals, there is evidence of alterations at the level of neurotransmitters, cytokines, and hormones, as well as changes in the nervous, immune, and endocrine systems. Currently, there is a greater knowledge of biological factors due to the advancement of research in this area. In particular, the important relationship between the central nervous system and the immune system has been described, showing that a disturbance in one system can be reflected in the other. [2]. Since the beginning of the pandemic, the prevalence of anxiety and depression has increased and, in some countries, has even doubled in value compared to previous years [3]. For this reason, stress is currently becoming a serious public health problem and is considered the cause of many pathologies and even aggravates pre-existing ones [4]. There is evidence that alterations occur at the level of neurotransmitters and their actions induce structural and functional changes in the central nervous system, which increase the risk of suffering from depression [5]. Several investigations during the 20th century managed to identify a decrease in the levels of these substances in the nervous system of patients with depression. This allows us to postulate that at least part of the disease is caused by a deficit in the transmission of these molecules [6]. Options for treating depression include medications, which will only achieve a modest response and a low remission rate [7]. The main goal of antidepressant treatment, whatever its modality, is to achieve complete remission of symptoms and allow patients to return to normalcy [8,9]. Antidepressant treatments to date have shown limited efficacy [10,11]. Most of the antidepressants that are prescribed continue to be developed based on their activity on the neurotransmission system [12]. Response rates and symptomatic remission are evident, although modest, and up to a third of patients will fail to achieve remission after multiple treatments [10,11]. Psychoeducation plays an important role in improving patient and medication adherence. Recent evidence also supports that lifestyle modification, including moderate exercise and eating habits, can help improve depression [13].

Depression is a multifactorial disease, and in this sense, current research shows how nutritional and physical activity habits could directly affect the symptomatology of depression [14,15]. In this line, physical activity improves physical fitness, self-esteem, anxiety, and depression in the current population [16]. There is sufficient evidence that demonstrates a bidirectional association between a sedentary lifestyle and depressive disorders; therefore, it is biologically evident that exercise can have antidepressant effects [17]. A recent study indicates that performing aerobic and resistance exercises produces a greater effect if they are combined in the same session than if they were performed separately [18]. On the other hand, it has been concluded that physical activity performed outdoors, in the natural environment, produces greater mental well-being than when performed indoors [19].

According to scientific evidence, there is an association between diet quality and mental health in terms of depression [20]. The foods ingested through the diet condition the intestinal microbiota, and this also influences mood and stress. Having good eating habits provides the recommended number of micronutrients, polyphenols, and fats that are related to the optimal functioning of the brain. This translates, in turn, into a good functioning of neurotransmitters and a decrease in neuroinflammation [21]. In addition, the appearance of different diseases, such as behavioral disorders, anxiety, and depression, could be partly explained by changes in microbiota concentrations [22]. In recent years, the importance of the so-called gut–brain axis has been highlighted, establishing the bidirectional role of the microbiota of the digestive tract and the central nervous system. This axis is postulated as a possible pathogenic basis of numerous neurological disorders with a great impact on mental health [23].

On the other hand, depression could also be related to oral health, since depressive patients tend to neglect their oral hygiene, leading to an increased risk of caries and periodontal disease [24]. Depressed patients, in general, have a higher risk of harmful habits such as tobacco, alcohol, and drugs—habits that are not beneficial for oral health. Many of these habits have hyposalivation properties. Many medications prescribed for depression cause xerostomia, which is the feeling that the mouth is dry, usually due to a decrease or absence of saliva that causes insufficient functioning of the salivary glands [25]. Decreased salivary flow results in increased caries, infections, gingivitis, and periodontal disease. Occasionally, mental illnesses, due to changes in mood, can worsen oral hygiene habits [26]. Previous evidence showed that different factors, such as nutritional habits, physical activity, and oral health factors, are factors that control the development of depression; therefore, considering that depression is a disease with an important behavioral basis, the understanding of descriptive behavior would allow for better prevention and treatment. For this reason, we carried out this research with the aim of studying the differences in behavioral, nutritional, dental, and physical activity patterns of the depressed group and the healthy control population. The initial hypothesis was that patients with depression would present lower physical activity, higher consumption of proinflammatory foods, and increased anxiety and loneliness than the healthy control population.

## 2. Materials and Methods

### 2.1. Participants

An intentional sampling participant recruitment was used to reach the final participants studied. Finally, 48 participants with depression (45.7 ± 12.0 years, 165.4 ± 7.8 cm, 71.7 ± 19.7 kg, and 26.1 ± 6.6 BMI) and 100 participants in the control group without any pathology or any medication (48.9 ± 7.9 years, 168.3 ± 11.6 cm, 67.9 ± 13,6 kg, and 23.9 ± 4.1 BMI) were analyzed. The inclusion criteria for the depression group were that participants presented 50 points or more on the Zung scale. The characteristics of the depression group are shown the Table 1.

### 2.2. Design and Procedure

To reach the study objective, an online questionnaire with items about the psychological profile, nutrition habits, physical activity, and oral profile was administered to the participants between February to September 2021. The specific questionnaires were as follows [25,27].

Psychological measures were performed by the following questionnaires: A short version of the Spanish version of the Big Five Inventory (Big Five Personality Trait Short Questionnaire) [28]. This scale analyses five factors of personality: neuroticism, extraversion, openness, kindness, and responsibility. The reduced version is composed of 10 items that are answered on a 5-point Likert scale, where 1 means completely disagree and 5 means completely agree. An example item is: “I see myself as a person who gets nervous easily”. A short version of the Spanish version of STAI (Spielberger State-Trait Anxiety Inventory) [29], composed of 6 items assessing anxiety that are answered on a 4-point Likert scale where 1 means not at all and 4 means very much, was used to measure anxiety. AAQ-II (Acceptance and Action Questionnaire II) [30] analyses the psychological inflexibility or experiential avoidance through 7 items, each answered by a 7-point Likert scale, where 0 means never true and 7 means always true. An example item is: “Emotions cause problems in my life”. High scores suggest that it is probable that there is current clinical distress. UCLA Loneliness Scale (Spanish version of the Three-Item Loneliness Scale) [31] assesses the measurement of loneliness. In the present study, we used a condensed version composed of three items, each answered by a 3-point Likert scale, where 1 is never and 3 is frequently. An example item is: “My interests and ideas are not shared by those around me”. PSS (Short Spanish version of the Perceived Stress Scale) [32] assesses the level of perceived stress in a one-month period. It is composed of 14 items answered on a 5-point Likert scale, meaning 0 = Never and 4 = Very often. An example item is: “In the last month, how often did you feel that you could not control important things in your life?”. The Spanish version of the Zung Depression Scale (Zung Self-Rating Depression Scale) [33] was used to measure depression. The Zung Depression Scale uses a self-applied scale for depression, which has a sensitivity and specificity greater than 80% and consists of 20 items formulated in positive and negative terms. Somatic and cognitive symptoms are highly relevant, with 8 items for each group. The scale also includes 2 items referring to mood and 2 to psychomotor symptoms. It is composed of 4 items, from a short time to most of the time. It is composed of 4 items, from a short time to most of the time. Spanish version of the Three-Item Loneliness Scale.

Nutrition Habits Measures. We used an adapted previously used questionnaire [34] to analyze eating habits and nutrition behaviors in the last 12 months in the population related to weekly consumption frequency of different food groups, including:

Fruit juices and nectars (250 mL);
Alcohol (Whiskey, rum, gin…) (50 mL approx.);
Beer (250 mL approx.);
Wine (50 mL approx.);
Soft drinks (coke, soda…) (250 mL approx.);
Energy drinks (250 mL approx.);
Coffee (250 mL approx.);
Tea (250 mL approx.);
Milk (250 mL approx.);
Fermented products (125 gr);
Pastries (1 portion);
Cookies–sweet cereals (30g-250mL);
Cheese (50 gr);
Eggs (1 piece);
Meat (150 gr);
Fish (150 gr);
Sausage/cold meat (150 gr);
Legumes (200 gr);
Rice (150 gr);
Pasta (150 gr);
Fruit (1 portion);
Raw vegetables (salad) (200 gr approx.);
Cooked vegetables (200 gr approx.);
Bread (50 gr approx.);
Whole-grain cereal (bread, rice, oat…);
Fast food (1 serving);
Protein shakes (300mL);
Vitamin supplements (1 capsule).

Each item ranged from 1 to 6, where 1 means “I do not consume”, 2 means “less than three times per week”, 3 means “three times or more per week”, 4 means “seven or more times per week”, 5 means “ten or more times per week”, and 6 means “more than thirteen times per week”.

We also evaluated the vitality during the week and at the end of the week in two questions, each answered by a 10-point Likert scale, where 0 means very low and 10 means very high. The question: ”Do you have a Migraine headache?” was answered by a 10-point Likert scale, where 0 means rarely and 10 means very often. For the question ”How satisfied do you feel with your weight? the answers were “completely satisfied”,” I would like to increase weight”, and” I would like to decrease weight”. For the question “How many glasses of water do you drink per day (250 mL)?”, the answers ranged from “0” to “more than 10”. For the question ”How many sugar spoons take per day? the answers ranged from “0” to “more than 5”. For the question ”How do you have post-meal digestion?” the answers were “I feel good normally”, “sometimes I feel hard, heavy digestion”,” very often I feel hard, heavy digestion”. For the question “In the last week, which type of feces did you have?” where 1 means “shard chunks”, 2 means “sausage shape”, 3 means “noodle form”, 4 means “snakes”, 5 means “soft chunks”, 6 means “soft chunks with undone limits” and 7 means “watery with no solid chunks”—we used the Bristol Scale [35].

Physical activity was evaluated with a questionnaire used in line with previous research [36,37] and was free to withdraw from the study at any time, which included the items:” How many average steps per day have you done in the last week?”, “Did you do any physical activity in the last 7 days?”, “If so, time in minutes of cyclic and/or aerobic activity (cycling, treadmill, Zumba) adding up all the sessions of the 7 days”, “If so, time in minutes of activity with self-loads (sit-ups, push-ups, squats…) or weights (gym machines, weights…) adding up all the sessions of the 7 days.” Average minutes of self-loads per session in the last week were measured on a self-perception scale, indicating the average minutes of self-loads the participant had taken in the last week.

Oral health was measured following a previously used questionnaire [36] consisting of 6 items related to oral health. For the first question: “How many times a day do you brush your teeth per day?”, the answers ranged from “none” to “more than four per day”. For the question “Do you smoke?”, answers ranged from “no” to “more than five cigarettes per day.” The rest of the 3 questions are “Do you suffer from gastritis or heartburn?”, “Does your mouth often feel dry as if it lacks saliva?”, and “Do you have dental sensibility?, and were answered by “yes,” “sometimes,” or “no.” For the last question: “How many days have you been sick last year (flu, cold…)?” This item was measured on a free choice scale, indicating the illness days of last year.

### 2.3. Statistical Analysis

The statistical analysis was carried out using the Statistical Package for the Social Sciences (SPSS) version 22.0 (SPSS Inc., Chicago, IL, USA). Descriptive statistics (mean and standard deviation) were calculated for each variable. The Kolmogorov–Smirnov normality test was used to test the homogeneity and distribution of each variable. The study variables presented a parametric distribution; then, to analyze group differences between the depression group and control group, an independent T-test was conducted. The significance level was *p* ≤ 0.05.

## 3. Results

According to the psychological profile, the depression group showed significantly higher levels on the Zung Depression Scale (*p* = 0.000), Perceives Stress Scale (*p* = 0.003), Spielberger State-Trait Anxiety Inventory (*p* = 0.003), UCLA Loneliness Scale (*p* = 0.001), Acceptance and Action Questionnaire II (*p* = 0.000), Big Five Factors Neuroticism (*p* = 0.030), and Big Five Factors Open to experience (*p* = 0.003). Lower levels of Big Five factors Extraversion (*p* = 0.030) were found in the depression group (Table 2).

Table 3 shows the results of the nutritional variables. The control group showed significantly higher week vitality (*p* = 0.000), vitality at the end of the week (*p* = 0.001), weekly frequency of juice (ml) (*p* = 0.011), weekly frequency of wine(ml) (*p* = 0.010), weekly frequency of coffee (ml) (*p* = 0.027), weekly frequency of fresh vegetables-salad (g) (*p* = 0.035), and Bristol Scale (*p* = 0.024) than the depression group.

Regarding oral health variables, the control group showed significantly lower levels of gastritis or heartburn (*p* = 0.000), dry mouth (*p* = 0.000), dental sensibility (*p* = 0.002), and illness days per year (*p* = 0.000) than the depression group (Table 4).

According to the physical activity variables, the control group showed a significantly higher level of physical activity in the last 7 days than the depression group (*p* = 0.030) (Table 5).

## 4. Discussion

The aim of the present study was to analyze the differences in behavioral, nutritional, dental, and physical activity patterns of the depressed and control population. The initial hypothesis was fulfilled since we found differences in behavior between the depression group and the control group.

Current lifestyles, especially in Western countries, are associated with the well-known diseases of civilization within which we can frame depression. Being able to understand what behavioral factors can modulate the incidence of this disease will allow us to carry out interventions at the level of prevention and interventions at the behavioral level that is more efficient and without secondary effects such as those of traditional pharmacological treatments [38,39,40,41].

Patients with depression presented significantly higher levels of depression and significantly lower values of extraversion. In general, extraversion is lower, and introversion is higher in depressed patients than in the general population [42,43]. Our results suggested that people who experienced higher stress had lower levels of psychological flexibility. This is consistent with previous literature that showed that higher perceived stress was associated with lower psychological flexibility [44]. We also found that lower psychological flexibility was associated with higher levels of anxiety and depression, which is in line with previous studies that suggested that lower psychological flexibility was associated with more depressive symptoms, more anxiety-related symptoms, and poorer psychological health in general [45]. In this line, several studies found how depression, stress, anxiety, loneliness, and neuroticism are parameters related to mental illness [46,47,48,49]. This fact could be related to a less adaptive profile and resilience in depressed people, in line with previous studies that revealed a significant reciprocal relationship between resilience and mental health status [50]. Resilience can help people protect themselves from various mental health conditions, such as depression and anxiety. People with pre-existing mental health problems can improve their coping ability by being resilient [51]; however, in this study, patients with depression showed a higher level of the open-to-experience personality factor. This result could be related to stressful life events that were significantly associated with openness to experience; openness to experience is partially involved in the relationship between stressful life events and depression [52].

Regarding nutritional habits, the depression group showed a significantly lower consumption of fresh vegetable salad and significantly higher consumption of soft drinks. This result was in line with previous studies that found that in the US adult population, consumption of sugar-sweetened beverages is associated with a 30% increased risk of depression [53]. In addition, other studies associated vegetable consumption with a 14% lower risk of depression [54]. Those studies provided further evidence that vegetable intake was associated with depression. The gut microbiota is altered in people with depression. Our microbiota needs fiber to feed itself and to be able to create the basic neurotransmitters of our brain, the lack of fiber is a biological cause of depression [55]. The findings support the current recommendation to increase vegetable intake to improve mental health, as they are rich in fiber [56]. A higher intake of fruits and vegetables and physical activity could reduce the risk of depression. The results of this study highlight the importance of physical activity and the consumption of fruits and vegetables to prevent depression [57]. It is also important to note that fish consumption provides essential omega-3 fatty acids EPA and DHA for brain structure and anti-inflammatory potential [58]. Depression, according to the gut–brain axis, is increasingly associated with dysbiosis, that is, with the alteration of our microbiota. Likewise, it is related to the chronic low-grade inflammatory state. Specifically, the intake of vegetables is relevant in reestablishing an adequate microbiota [59]. Dysbiosis and inflammation of the gut have been linked to depression [60]. In fact, sugar consumption increases inflammation, and therefore there is a relationship between inflammation and depression [61].

On the other hand, a higher incidence of migraine was found in the depression group. Previous studies found that there was an association between migraine and depression [62] since almost 80% of people who suffer from migraine at one time or another in their life have depression [63]. On the other hand, it was found that in the serum of depressed patients, there was an increase in the level of inflammatory cytokines in the brain [64]. This could be related to the low-grade chronic inflammation that we talked about earlier, related to nutritional aspects. There is evidence that suggests inflammation as a possible etiological factor of mood disorders [65]; it was even shown how diets with proinflammatory foods could change the perception that a subject may have of danger—a very important factor to take into account to modulate the final behavior of a person [66]. Thus, some diseases of the digestive system that cause inflammation and irritation, such as gastritis and postprandial digestion, also with higher levels in the depression group, worsen when stress or anxiety increases [67]. This is consistent with a study that found that people who have a diet rich in inflammatory ingredients such as processed foods, trans fats, or alcohol are more likely to experience depression [68].

We also found lower levels of weekly vitality in the depression group—a result that could be related to their higher BMI and sedentary habits. Previous studies demonstrated how moderate-intensity functional training was effective in improving quality of life, vitality, and depression [69]. The depression group showed higher levels of gastritis, dry mouth, and dental sensitivity, parameters related to poorer oral health. Patients with mental disorders are subject to a greater number of risk factors for oral diseases [70]. The danger of increased periodontal diseases in depressive patients is linked to mood changes that lead them to abandon oral hygiene habits [71]. Our results agreed with previous studies showing how the composition of the microbiome inside the mouth is associated with depression in adults and supported an important role of alterations in the brain–gut microbiome axis in the origin of depression [72]. Dry mouth was linked in previous studies with depressive symptoms that were significantly associated with dry mouth sensation [73]; however, it should be noted that dry mouth is also a common side effect of many types of antidepressant medications that are widely used for the treatment of this disease [74]. Regarding tooth sensitivity, the depression group showed higher levels. As we have described previously, there are studies that associate depression with inadequate oral health, and this affects the periodontal status; in turn, the oral immune system is also affected, causing inflammation and increasing dental sensitivity [75].

Another factor related to the quality of life evaluated is the number of sick days per year, which was also higher in the depression group. This result is in line with numerous studies that found that people with depression were at increased risk of certain physical illnesses [76]. Sick days in depressive patients are related to precarious levels of health, hyperactivity of the nervous system, and low psychological profile (low resilience, coping, high anxiety…). In short, depressive patients have a higher risk of infections due to this hyperactivity of their nervous system [77].

Regarding physical activity, the current data revealed how both groups presented low levels of physical activity, being lower in the depression group, results probably affected by the pandemic situation. We all know that during the COVID pandemic, having adequate levels of physical activity during this time to ensure optimal health was difficult due to the strict lockdown. Sedentary habits, including home confinement, resulted in increased physical inactivity and sedentary behavior [78], and thus, more mood disorders. The values in the physical activity variables presented in the analyzed sample were lower than the recommendations of the health-promoting institutions. In recent years, several studies have found a lower level of physical activity among people with depression or depressive symptoms [79]. There are studies that found a significant decrease in anxiety and depression levels in adults who performed regular physical–recreational activities [80]. Performing some physical activity in leisure time decreases the probability of manifesting depressive symptoms [81]. It is very important to promote physical activity to reduce depression in our society. Finally, physical activity is one of the cheapest non-pharmacological treatments with the greatest impact on public health. Physical activity should be integrated into daily life at all ages [57]. There are WHO guidelines and recommendations for different age groups and specific population groups on how much physical activity is needed for good health [82]. On the other hand, it is important to highlight the anti-inflammatory effect that physical activity has, as well as the positive effect at the mental level that it has in patients with depression [83]. Moderate physical activity acts as an anti-inflammatory and opens the door to the treatment of chronic pathologies such as depression [84], and if it is also combined with nutritional and psychological intervention, improvements can be achieved in short periods of time [85].

The main limitation of this research was the low number of participants analyzed, but the restriction due to the COVID-19 pandemic limited access to a larger sample. Overall, we found that the quality of life of people during the COVID-19 pandemic affected the anxiety and depression of the population [86]. The results of this study open a new multidisciplinary field in the study of depression. The combination of recommendations on oral health, nutritional habits, and physical activity could be an effective tool to reduce the risk of depression in future generations. Finally, future prospective observational studies are needed to establish the causal role of physical activity, nutritional habits, and oral health more clearly in the development and progression of depression. The mechanisms by which oral health, nutritional and physical activity habits can cause a reduction in depression are still not sufficiently clear. Additional scientific evidence is still required to inform the efficacy and specificity of sedentary behavior recommendations for clinical practice and for public health policies aimed at reducing the burden of depression in the current population. Furthermore, economic status was not included in the questionnaires and could be considered an important issue to consider in future research, as highlighted by previous authors [87]. For practical applications in the future, we find that behavioral habit analysis can be considered a useful tool to prevent immediate and long-term risks of depression in the population. Knowledge of dietary factors could be used by health institutions to implement multidisciplinary interventions to reduce bad sedentary habits among people; therefore, comprehensive interventions in the population are crucial to acquire the appropriate knowledge, beliefs, skills, and attitudes that help shape a healthy lifestyle and reduce depression in the future.

In summary, we found that the depression group presented significantly higher anxiety, depression, loneliness, perceived stress, psychological inflexibility, open-to-experience personality factor, consumption of soft drinks, migraine, and post-meal digestion; the depression group also had significantly lower consumption of juice, wine, coffee, and fresh vegetable salad, and lower vitality than the control group. In addition, patients with depression also presented poor dental health, presenting higher rates of gastritis, dry mouth, dental sensitivity, and sick days per year. Both groups presented a pattern of low physical activity. This study shows the importance of promoting physical activity and healthy nutritional and oral health habits to reduce the high levels of depression in our society. Increased physical activity is associated with a lower risk of depression. Increased physical activity along with a healthy diet rich in fruits and vegetables are important, safe, effective, and cost-effective healthy behaviors that can help the general population maintain good physical and mental health and reduce the risk of depression. This study highlights the importance of exercising more and having better nutritional and oral health habits. These simple changes in healthy habits and behavior can have a positive impact and reduce public health costs; however, governments must provide social welfare services, health insurance, educational programs, and outdoor social activities for the population. The results of this study open a new multidisciplinary field in the treatment of depression, which is so present in the world today.

## 5. Conclusions

The results of this study highlight the behavioral differences in patients with depression. We found that depression patients presented a less adaptative psychological profile, lower ingestion of foods with fiber, vitamins, and trace elements, higher digestive tract problems, decreased vitality, and poor dental health than the control healthy population. In addition, the depression group presented a low physical activity compared to the control group.

## Figures and Tables

**Table 1:** Characterization of depression group.

Severity	Clinical Form	Therapeutical Control	Compliance
Moderate and severe	Major depression	Selective serotonin reuptake inhibitors (SSRIs)	>80%

**Table 2:** Results of psychological variables analyzed.

					Confidence	Interval
Variable	Depression Group	Control Group	T	*p*	Lower	Upper
PSS4	14.9 ± 4.0	12.8 ± 4.0	−2.994	0.003	−3.51	−0.72
STAI	14.9 ± 4.0	12.8 ± 4.0	−2.994	0.003	−3.51	−0.72
UCLA	4.9 ± 1.7	4.1 ± 1.3	−3.430	0.001	−1.40	−0.38
AAQII	26.4 ± 11.4	19.5 ± 9.1	−3.921	0.000	−10.32	−3.40
Extraversion	5.1 ± 1.6	5.8 ± 1.7	2.187	0.030	0.06	1.24
Agreeableness	7.0 ± 1.8	6.7 ± 1.4	−0.966	0.336	−0.83	0.28
Conscientiousness	7.3 ± 2.1	7.8 ± 1.5	1.715	0.088	−0.08	1.10
Neuroticism	7.1 ± 1.9	6.0 ± 1.9	−3.398	0.001	−1.83	−0.48
Open to experience	8.1 ± 1.5	7.2 ± 1.8	−3.013	0.003	−1.51	−0.31

PSS4—Perceives Stress Scale; STAI—Spielberger State-Trait Anxiety Inventory; UCLA—UCLA Loneliness Scale; AAQII—Acceptance and Action Questionnaire II; Big Five Factors (Extraversion, Agreeableness, Conscientiousness, Neuroticism, and Openness to experience).

**Table 3:** Nutritional variables.

					Confidence	Interval
Variable	Depression Group	Control Group	T	*p*	Lower	Upper
Water glasses per day (250mL)	2.6 ± 1.5	2.7 ± 1.4	0.098	0.922	−0.46	0.51
Week Vitality (0–10)	6.3 ± 2.2	7.7 ± 1.4	4.870	0.000	0.87	2.05
Vitality at the end of the week (0–10)	5.1 ± 2.7	6.6 ± 2.4	3.522	0.001	0.67	2.40
Migraine headache (0–10)	4.7 ± 3.5	2.1 ± 2.9s	−4.708	0.000	−3.61	−1.48
Weekly frequency of juice (250 mL)	1.5 ± 0.8	2.1 ± 1.2	2.991	0.003	0.19	0.95
Weekly frequency of distilled alcohol (50 mL)	1.2 ± 0.6	1.3 ± 0.5	0.456	0.649	−0.14	0.23
Weekly frequency of beer (250 mL)	1.8 ± 0.8	1.9 ± 1.0	0.881	0.380	−0.19	0.49
Weekly frequency of wine (50 mL)	1.4 ± 0.6	1.7 ± 0.7	2.610	0.010	0.08	0.58
Weekly frequency of soft drink (250 mL)	1.8 ± 1.2	1.4 ± 0.8	−2.131	0.035	−0.70	−0.03
Weekly frequency of energy drink (250 mL)	1.1 ± 0.3	1.1 ± 0.4	0.144	0.886	−0.12	0.14
Weekly frequency of coffee (250 mL)	2.6 ± 1.5	2.7 ± 1.5	2.236	0.027	0.07	1.17
Weekly frequency of tea (250 mL)	1.7 ± 1.1	1.8 ± 1.2	0.517	0.606	−0.32	0.54
Weekly frequency of milk (250 mL)	2.5 ± 1.4	2.4 ± 1.4	−0.202	0.840	−0.55	0.45
Weekly frequency of fermented milk (250 mL–125 g)	2.3 ± 1.7	2.7 ± 1.3	1.773	0.078	−0.48	0.88
Weekly frequency of sweets (250 mL–30 g)	1.6 ± 0.7	1.8 ± 1.0	1.118	0.265	−0.15	0.53
Weekly frequency of cookies–sweet cereals (250 mL–30 g)	1.7 ± 0.9	2.0 ± 1.2	1.396	0.165	−0.12	0.68
Weekly frequency of cheese (250 mL–50 g)	2.5 ± 1.1	2.5 ± 1.1	−0.242	0.809	0.20	0.35
Weekly frequency of eggs (1 unit)	2.6 ± 0.8	2.6 ± 0.8	0.392	0.695	0.15	0.35
Weekly frequency of meat (150 g)	2.8 ± 0.9	2.7 ± 1.0	−0.486	0.628	−0.41	0.25
Weekly frequency of fish (150 g)	2.4 ± 0.7	2.5 ± 0.8	0.581	0.562	−0.21	0.38
Weekly frequency of sausage (50 g)	2.1 ± 1.2	2.1 ± 1.0	−0.334	0.739	−0.44	0.31
Weekly frequency of legumes (200 g)	2.4 ± 0.9	2.4 ± 0.8	−0.071	0.943	−0.30	0.28
Weekly frequency of rice (150 g)	2.3 ± 0.8	2.2 ± 0.7	−0.766	0.445	−0.36	0.16
Weekly frequency of pasta (150 g)	2.2 ± 0.8	2.1 ± 0.7	−0.723	0.471	−0.34	0.16
Weekly frequency of fruits (1 unit)	3.1 ± 1.5	3.6 ± 1.5	1.809	0.073	−0.045	1.02
Weekly frequency of fresh vegetables–salad (200 g)	2.7 ± 1.1	3.2 ± 1.3	2.127	0.035	0.34	0.92
Weekly frequency of cooked vegetables (200 g)	2.8 ± 1.0	3.1 ± 1.3	1.102	0.272	−0.19	0.67
Weekly frequency of bread (50 g)	2.8 ± 1.2	3.1 ± 1.4	1.299	0.196	−0.16	0.79
Weekly frequency of whole grains-rice-oat (250 mL)	2.2 ± 1.3	2.1 ± 1.1	−0.766	0.445	−0.60	0.26
Weekly frequency of fast food-pizza-hamburger (1 unit)	1.7 ± 0.7	1.6 ± 0.7	−0.456	0,649	−0.30	0.19
Weekly frequency of protein shakes (300 mL)	1.1 ± 0.5	1.1 ± 0.4	−0.295	0.768	−0.18	0.13
Weekly frequency of vitaminic supplements (1 capsule)	1.7 ± 1.3	1.4 ± 0.9	−1.813	0.072	−0.71	0.03
Daily coffee spoons of sugar (number)	1.4 ± 0.5	1.4 ± 0.6	−0.589	0.557	−0.28	0.15
Post-meal digestion (1–5)	1.7 ± 0.8	1.3 ± 0.5	−3.714	0.000	−0.64	−0.19
Bristol Scale	3.2 ± 1.5	3.7 ± 1.1	2.280	0.024	0.07	0.94

**Table 4:** Oral health variables.

					Confidence	Interval
Variable	Depression Group	Control Group	T	*p*	Lower	Upper
Daily toothbrushing	2.4 ± 0.9	2.6 ± 0.8	1.545	0.125	−0.06	0.53
Smoker	1.5 ± 1.0	1.4 ± 0.9	−0.645	0.520	−0.45	0.23
Gastritis or heartburn	1.8 ± 0.7	1.3 ± 0.6	−4.244	0.000	−0.66	−0.24
Dry mouth	1.9 ± 0.8	1.4 ± 0.7	−3.673	0.000	−0.72	−0.21
Dental sensibility	1.9 ± 0.9	1.5 ± 0.7	−3.089	0.002	−0.68	−0.15
Illness days per year	8.3 ± 16.5	1.6 ± 3.3	−3.899	0.000	−10.12	−3.31

**Table 5:** Physical activity variables.

					Confidence	Interval
Variable	Depression Group	Control Group	T	*p*	Lower	Upper
Daily Steps last week	7870.8 ± 5750.1	10,899.5 ± 13,078.5	1.296	0.198	−1600.62	7657.98
Physical Activity last 7 days (h)	2.4 ± 0.9	2.7 ± 0.7	2.196	0.030	0.03	0.57
Aerobic activity last 7 days (min)	134.5 ± 165.1	199.4 ± 280.1	1.225	0.223	−40.11	169.88
Self-loading last 7 days (min)	43.7 ± 98.6	60.0 ± 88.1	0.852	0.396	−21.58	54.11

## Data Availability

All data are in the manuscript.
